# Supplementary material for: Prognostic Significance of Volumetric Parameters Based on FDG PET/CT in Patients with Lung Adenocarcinoma Undergoing Curative Surgery
Source: Cancers (Basel). 2023 Sep 1;15(17):4380. doi: 10.3390/cancers15174380 (PMC10486443; doi:10.3390/cancers15174380)
Supplement: Supplementary file 1 [file cancers-15-04380-s001.zip › cancers-2583856-supplementary.docx]

**SUPPLEMENTARY TABLES**

**Supplementary Table S1. Image acquisition protocols**

| **Protocols** | **Instrument** | |
| --- | --- | --- |
|  | **Discovery LS** | **Discovery STE** |
| Fasting | At least six hours | |
| Blood glucose level | Less than 200 mg/dL | |
| Acquisition time | 60 minutes | |
| Dose | 5.0 MBq/kg | |
| CT voltage | 140 keV | |
| CT current | 40–120 mA | 30–170 mA |
| Emission scan | 4 minutes per frame in 2-dimensional mode | 2.5 minutes per frame in 3-dimensional mode |
| Reconstruction | OSEM algorithm with 28 subsets and 2 iterations | OSEM algorithm with 20 subsets and 2 iterations |
| Matrix size | 128 × 128 | |
| Voxel size | 4.3 × 4.3 × 3.9 mm | 3.9 × 3.9 × 3.3 mm |

OSEM, ordered-subsets expectation maximization**.**

**Supplementary Table S2. Univariate Cox regression analysis for disease-free survival in the patients with operative lung adenocarcinoma**

| **Variable** | **Categories** | **HR (95% CI)** | **P** | **P of log-rank test** |
| --- | --- | --- | --- | --- |
| Sex | Female vs. Male | 1.241 (0.942-1.636) | 0.125 | 0.124 |
| Age | <58 |  |  | 0.064 |
|  | 58~67 | 0.886 (0.625-1.255) | 0.494 |  |
|  | 67≤ | 1.303 (0.939-1.808) | 0.113 |  |
| Age (continuous) |  | 1.009 (0.995-1.023) | 0.204 | - |
| Location | Left vs. Right | 1.056 (0.801-1.391) | 0.699 | 0.700 |
| Adjuvant therapy | No vs. Yes | 2.041 (1.549-2.689) | < 0.001 | < 0.001 |
| Histological grade | WD/MD vs. PD | 1.165 (0.873-1.556) | 0.300 | 0.299 |
| EGFR mutation | No vs. Yes | 0.999 (0.759-1.315) | 0.992 | 0.991 |
| ALK mutation | No vs. Yes | 1.205 (0.760-1.913) | 0.428 | 0.427 |
| Pathological T stage | T_mi_/T1 |  |  | 0.013 |
|  | T2 | 1.549 (1.137-2.112) | 0.006 |  |
|  | T3 | 1.526 (0.979-2.377) | 0.062 |  |
|  | T4 | 1.990 (1.121-3.531) | 0.019 |  |
| Pathological N stage | N0 |  |  | < 0.001 |
|  | N1 | 1.762 (1.203-2.582) | 0.004 |  |
|  | N2 | 3.545 (2.596-4.842) | < 0.001 |  |
| Pathological substage | IA |  |  | < 0.001 |
|  | IB | 1.844 (1.125-3.022) | 0.015 |  |
|  | IIA | 1.971 (0.993-3.910) | 0.052 |  |
|  | IIB | 2.114 (1.338-3.342) | 0.001 |  |
|  | IIIA | 4.852 (3.210-7.333) | < 0.001 |  |
|  | IIIB | 4.720 (2.172-10.258) | < 0.001 |  |
| Pathological stage | I |  |  | < 0.001 |
|  | II | 1.614 (1.122-2.322) | 0.010 |  |
|  | III | 3.745 (2.702-5.191) | < 0.001 |  |
| SUVmax | Low vs. High | 2.147 (1.533-3.007) | < 0.001 | < 0.001 |
| SUVmax (continuous) |  | 1.050 (1.023-1.078) | < 0.001 | - |
| SUVmean | Low vs. High | 1.885 (1.422-2.500) | < 0.001 | < 0.001 |
| SUVmean (continuous) |  | 1.240 (1.126-1.366) | < 0.001 | - |
| MTV | Low vs. High | 1.901 (1.429-2.530) | < 0.001 | < 0.001 |
| MTV (continuous) |  | 1.009 (1.004-1.013) | < 0.001 | - |
| TLG | Low vs. High | 1.999 (1.515-2.639) | < 0.001 | < 0.001 |
| TLG (continuous) |  | 1.001 (1.000-1.002) | 0.008 | 0.007 |

HR, hazard ratio; CI, confidential interval; SUVmax, maximum standardized uptake value; SUVmean, mean standardized uptake value; MTV, metabolic tumor volume; WD, well-differentiated; MD, moderately-differentiated; PD, poorly-differentiated.

**Supplementary Table S3. Multivariate Cox regression analysis for overall survival including SUVmax and SUVmean in the patients with operative lung adenocarcinoma.**

| **Variable** | **Categories** | **SUVmax** | | **SUVmean** | |
| --- | --- | --- | --- | --- | --- |
|  |  | **HR (95% CI)** | **P** | **HR (95% CI)** | **P** |
| **Sex** | Female vs. Male | 1.509 (0.936-2.432) | 0.091 | 1.439 (0.893-2.319) | 0.136 |
| Age | <58 |  |  |  |  |
|  | 58~67 | 1.582 (0.827-3.029) | 0.166 | 1.599 (0.835-3.063) | 0.157 |
|  | 67≤ | 3.267 (1.779-6.000) | < 0.001 | 3.351 (1.826-6.148) | < 0.001 |
| Adjuvant therapy | No vs. Yes | 0.752 (0.428-1.321) | 0.321 | 0.748 (0.426-1.312) | 0.311 |
| Histological grade | WD/MD vs. PD | 1.275 (0.802-2.028) | 0.305 | 1.241 (0.764-2.016) | 0.383 |
| EGFR mutation | No vs. Yes | 0.601 (0.365-0.988) | 0.045 | 0.587 (0.358-0.965) | 0.036 |
| Pathological stage | I |  |  |  |  |
|  | II | 2.491 (1.218-5.096) | 0.012 | 2.434 (1.182-5.013) | 0.016 |
|  | III | 5.260 (2.609-10.604) | < 0.001 | 5.178 (2.545-10.537) | < 0.001 |
| SUVmax | Low vs. High | 1.676 (0.896-3.134) | 0.106 |  |  |
| SUVmean | Low vs. High |  |  | 1.466 (0.900-2.387) | 0.124 |

HR, hazard ratio; CI, confidential interval; SUVmax, maximum standardized uptake value; SUVmean, mean standardized uptake value; WD, well-differentiated; MD, moderately-differentiated; PD, poorly-differentiated.

**Supplementary Table S4. Multivariate Cox regression analysis for disease-free survival including MTV and TLG in the patients with operative lung adenocarcinoma.**

| **Variable** | **Categories** | **MTV** | | | **TLG** | |
| --- | --- | --- | --- | --- | --- | --- |
|  |  | **HR (95% CI)** | **P** | **HR (95% CI)** | | **P** |
| Adjuvant therapy | No vs. Yes | 0.962 (0.646-1.432) | 0.848 | 0.956 (0.642-1.422) | | 0.823 |
| Pathological stage | I |  |  |  | |  |
|  | II | 1.452 (0.904-2.331) | 0.123 | 1.372 (0.851-2.212) | | 0.195 |
|  | III | 3.444 (2.146-5.525) | < 0.001 | 3.348 (2.091-5.360) | | < 0.001 |
| MTV | Low vs. High | 1.433 (1.056-1.945) | 0.021 |  | |  |
| TLG | Low vs. High |  |  | 1.582 (1.173-2.134) | | 0.003 |

HR, hazard ratio; CI, confidential interval; MTV, metabolic tumor volume; TLG, total lesion glycolysis.

**Supplementary Table S5. Multivariate Cox regression analysis for disease-free survival including SUVmax and SUVmean in the patients with operative lung adenocarcinoma.**

| **Variable** | **Categories** | **SUVmax** | | | **SUVmean** | | |
| --- | --- | --- | --- | --- | --- | --- | --- |
|  |  | **HR (95% CI)** | **P** | **HR (95% CI)** | | **P** |  |
| Adjuvant therapy | No vs. Yes | 0.981 (0.660-1.457) | 0.924 | 0.967 (0.650-1.441) | | 0.871 |  |
| Pathological stage | I |  |  |  | |  |  |
|  | II | 1.500 (0.947-2.377) | 0.084 | 1.487 (0.936-2.363) | | 0.093 |  |
|  | III | 3.390 (2.134-5.386) | < 0.001 | 3.495 (2.189-5.580) | | < 0.001 |  |
| SUVmax | Low vs. High | 1.756 (1.246-2.475) | 0.001 |  | |  |  |
| SUVmean | Low vs. High |  |  | 1.550 (1.159-2.072) | | 0.003 |  |

HR, hazard ratio; CI, confidential interval; SUVmax, maximum standardized uptake value; SUVmean, mean standardized uptake value.

**Supplementary Table S6. Univariate Cox regression analysis of overall survival according to conventional stage and proposed new stage including MTV the patients with operative lung adenocarcinoma.**

| **Variable** | **Categories** | **Hazard ratio** | **95% confidence interval** | **P** |
| --- | --- | --- | --- | --- |
| Pathological stage | I |  |  |  |
|  | II | 2.375 | 1.288-4.378 | 0.006 |
|  | III | 4.461 | 2.557-7.783 | < 0.001 |
| Proposed new stage by MTV | I (I with low MTV + II with low MTV) |  |  |  |
|  | II (I with high MTV + II with high MTV + III with low MTV) | 3.128 | 1.858-5.265 | < 0.001 |
|  | III (III with high MTV) | 6.703 | 3.731-12.042 | < 0.001 |

MTV, metabolic tumor volume.
